# iTRAQ-Based Proteome Profiling of Differentially Expressed Proteins in Insulin-Resistant Human Hepatocellular Carcinoma

**DOI:** 10.3389/fcell.2022.836041

**Published:** 2022-02-25

**Authors:** Jing Yan, Bei Xie, Ye Tian, Li Huang, Shuli Zou, Zhiheng Peng, Zhuan Liu, Linjing Li

**Affiliations:** ^1^ Department of Clinical Laboratory Center, The Second Hospital of Lanzhou University, Lanzhou, China; ^2^ Department of Medical Laboratory Animal Science, School of Basic Medical Sciences, Lanzhou University, Lanzhou, China; ^3^ Department of Pediatric Nephrology, The Second Hospital of Lanzhou University, Lanzhou, China; ^4^ Department of Medicine, Brookdale University Hospital Medical Center, Brooklyn, NY, United States

**Keywords:** proteome profiling, proteins, iTRAQ, PRM, insulin resistant, hepatocellular carcinoma

## Abstract

Recently, the incidences of insulin resistance (IR) and IR-related complications have increased throughout the world, which also associate with poor prognosis in hepatocellular carcinoma (HCC). Numerous studies had been focused on the role of IR in tumorigenesis and prognosis of HCC. The proteomic analysis of IR related hepatocellular carcinoma had not been reported by now. In the present study, 196 differentially expressed proteins (DEPs) were identified between insulin resistant HepG2 cells and their parental cells, of which 109 proteins were downregulated and 87 proteins were upregulated. Bioinformatics analysis indicated that these DEPs were highly enriched in process of tumorigenesis and tumor progression. PPI network analysis showed that SOX9, YAP1 and GSK3β as the key nodes, were involved in Wnt and Hippo signaling pathways. Survival analysis revealed that high expression of SOX9 and PRKD3 were strongly associated with reduced patient survival rate. parallel reaction monitoring (PRM) and Western blot analysis were applied to verify the protein level of these four key nodes mentioned above, which showed the same trend as quantified by isobaric tags for relative and absolute quantitation (iTRAQ) and confirmed the reliability of our Proteome Profiling analysis. Our results indicated that IR related dysregulation of protein expression might participated in tumorigenesis and malignant phenotype of hepatocarcinoma cells.

## Introduction

Insulin resistance (IR), which is clinically characterized as the inability of insulin to increase glucose uptake and utilization in individuals, is a common condition that has close relationship with metabolic syndrome (Kong et al., 2018). Recently, IR and IR-related complication have attracted extensive interest because of increasing incidence throughout the world (Ye, 2013). In the past few decades, considerable studies have revealed that IR is involved in the development and progression of various tumors and is associated with poor prognosis, especially in hepatocellular carcinoma (HCC) (Reaven, 1988; Li et al., 2017). As the main target organ of insulin, any pathological processes such as inflammation or neoplasia occur in the liver, will block the insulin signaling pathway and cause IR, which has been shown to be an independent risk factor accelerating the progression of HCC (Farrell, 2014). For instance, that hepatitis C virus (HCV) had been reported to induce IR by disturbing the insulin signaling pathway in an animal model (Koike, 2006) and IR could start at the early stage of infection and facilitated development of hepatic fibrosis and HCC (Mohamed et al., 2011). In addition, insulin could also induce proliferation and resist apoptosis of HCC through multiple pathways (Törnkvist et al., 1994; Kang et al., 2003), thus affecting the growth of liver cancer and survival of patient with HCC.

It is well known that any defect of insulin and its related signal transduction such as PI3K/AKT signaling pathway could lead to insulin resistance (Gao et al., 2016; Chaudhury et al., 2017). Also, IR could affect several signaling pathways which were related to tumor. For instance, IR contributes to tyrosine phosphorylation of insulin receptor tyrosine kinase substrate (IRTKS) and stimulates the growth of hepatocytes via AKT and mTOR signaling pathways (Wu et al., 2019). It is reported that the mTOR pathway is activated in the liver of obese rats, which is related with obesity-induced IR (Khamzina et al., 2005). A large number of researches had focused on the role of IR in occurrence and prognosis of HCC (Khamzina et al., 2005; Gao et al., 2016; Wu et al., 2019; Lei et al., 2020), however the relationship between chemotherapy efficacy and IR in HCC has been rare by now.

We previously proved that IR contributed to multidrug resistance (MDR) in HepG2 cells via upregulation of Bcl-2 and P-gp expression and activation of the *p*-ERK signaling pathway (Liu et al., 2016). Beyond that, we also found that autophagy played a crucial role in IR-mediated chemo-resistance in HCC by regulating the endoplasmic reticulum stress (Li et al., 2018), and many differentially expressed miRNAs (miR-134-5p, miR-5195-3p) and predicted target genes (*YAP1, SOX9, TPM4*) might be related to IR-mediated chemoresistance in HCC (Li et al., 2020). Furthermore, Kang et al. (2003) also declared that insulin could inhibit apoptosis by reducing oxidative stress via PI3K- and ERK-dependent signaling pathways in HepG2 cells.

Proteomic analysis is a valuable approach to discover the biomarkers and drug targets. However, the protein panorama in IR HCC had not been reported so far. Recently, the technology of isobaric tags for relative and absolute quantitation (iTRAQ) and parallel reaction monitoring (PRM) based quantitative proteomics have been wildly used to track proteomic changes. In the present study, iTRAQ and PRM combined with liquid chromatography tandem mass spectrometry (LC-MS/MS) were used to determine the protein abundance distribution, and to identify the differentially expressed proteins (DEPs) between insulin resistant HepG2 cells and its parental cells. Bioinformatic data mining was applied to explore functions and signaling pathways involved of the DEPs. At the same time, Western blot analysis was also carried out on individual DEPs of interest to confirm the results obtained by iTRAQ and PRM LC-MS/MS. Considering IR could contribute to MDR in hepatoma cells (Liu et al., 2016), these data we reported might provide novel insights for malignant phenotype related proteins in HCC.

## Materials and Methods

### Cell Culture

HepG2 cells were obtained from American Type Culture Collection (ATCC HB-8065, Rockville, MD, USA) and cultured with Dulbecco’s modified eagle medium (DMEM) and 10% fetal bovine serum (FBS) in the laboratory at temperature of 37 C with 5% CO_2_. HepG2 cells with IR resistance (HepG2/IR) were established according to a previously reported method. Firstly, HepG2 cells were cultured in serum-free DMEM for 6 h. After the cells were synchronized, they were treated with insulin at a concentration of 0.5 μM for 72 h on each cell culture flask (Liu et al., 2016). Authentication of the cells was performed by short tandem repeat (STR) analysis. Cells were monitored for *mycoplasma* contamination by MycoAlert^®^ *Mycoplasma* Detection Kit (Lonza, Rockland, MA, USA).

### iTRAQ Labeling, Strong Cation Exchange (SCX) Chromatography and LC-MS/MS Analysis

The protein from each sample was extracted and quantified using the BCA Protein Assay Kit (Bio-Rad, USA). For digestion, the filter-aided sample preparation (FASP) procedure was performed. iTRAQ reagent was used to label peptide mixture of each sample, then they were lysed by SCX chromatography. The collected fractions were concentrated by vacuum centrifugation and desalted on C18 Cartridges and analyzed by LC-MS/MS. The instrument was run with peptide recognition mode enabled. Protein identification and quantification were analyzed with Proteome Discoverer 1.4 (Thermo Scientific) and Mascot 2.2 (Matrix Science, UK). Protein screening criteria was set to less than 0.01of false discovery rate (FDR). The DEPs were screened by criteria with changes greater than 1.2 times or less than 0.83 times and *p* value less than 0.05.

### COG and KOG Function Annotation

To annotate Cluster of Orthologous Groups (COG) and orthologous groups (KOG), the Fasta file of COG database was downloaded and the blast database of COG was built. Then, the protein sequences were aligned to the COG database using Blastp software version of 2.12.0, COG proteins with similar sequences were obtained. The corresponding function is classified according to the COG ID. The annotation methods of KOG were the same as COG.

### Function Analysis

Gene ontology (GO) and Kyoto Encyclopedia of Genes and Genomes (KEGG) were performed to analyze the DEPs. GO terms were mapped by using Uniprot database and Blast2GO (Conesa et al., 2005) (https://www.blast2go.com/) for functional annotation. Kobas 3.0 (Bu et al., 2021) online software ( http://kobas.cbi.pku.edu.cn/anno_iden.php ) was adopted for KEGG to identify the enriched functions and pathways that might be involved. The Fisher’ exact test was applied to GO and KEGG enrichment analysis. For gene set enrichment analysis (GSEA), we used the R software package “clusterprofiler” and molecular signatures database (http://www.gsea-msigdb.org/gsea/downloads.jsp) to evaluate relevant pathways and molecular mechanisms. Functional categories and pathways were considered as significant when *p* values were under a threshold of 0.05.

### Protein-Protein Interaction Network Analysis

The DEPs were mapped by the STRING database to obtain the interaction information with the other DEPs. Protein interaction pairs with a comprehensive score greater than 0.4 (medium) were screened and protein-protein interaction network was analyzed online (https://string-db.org/). Subsequently, the Cytoscape software (http://www.cytoscape.org/, version 3.8.2) was employed to visualize the protein co-effection and then MCODE (Molecular Complex Detection) plug-in in Cytoscape were used to screened the key modules (Node Score Cutoff = 0.2, K-Core = 2, Max. Depth = 100).

### Targeted Protein Quantification by PRM and Western Blot

For PRM analysis, protein treatment was performed in the same way as in the iTRAQ experiment. The peptide information was imported into the software Xcalibur for PRM method setting. Chromatographic separation was carried out using high performance liquid chromatography (HPLC) system. The samples separated by HPLC were subjected to PRM mass spectrometry using a Q-Active HF mass spectrometer (Thermo Scientific). Finally, Skyline 3.5.0 software was used to analyze the original PRM files.

For Western blot assay, the procedure of cell lysate, protein quantification, separation and transfer were performed as before (Li et al., 2018). Then, membranes were blocked in skim milk at room temperature (RT) and probed overnight with primary antibodies including anti-YAP1 (1:2000; cat. no. ab109307; Abcam), anti-SOX9 (1:2000; cat. no. ab185966; Abcam), anti-PRKD3 (1:2000; cat. no. ab252982; Abcam), anti-GSK-3β (1:2000; cat. no. ab32391; Abcam), and anti-β-actin (1:1,000; cat. no. TA-09; Zhongshan Jinqiao Bio-Technology Co., Ltd.) as the internal reference. After incubation with secondary antibody at RT for 1.5 h, the PVDF membranes were incubated with ECL reagent. The images were captured in imaging system (GE Healthcare Life Sciences).

### Statistical Analyses

All experimental data were presented as means ± standard and repeated for at least three times. SPSS16.0 statistical software was conducted to compare the differences among treatments. For survival analysis, R software package ‘survival’ to integrate survival time was employed to evaluate the prognostic significance of selected proteins. Kaplan-Meier curves were plotted and a log-rank test was used to check the difference expression in overall survival between normal and tumor groups. *p* < 0.05 was considered statistically significant. The data were representative of three independent experiments performed in triplicate.

## Result

### Protein Profiling

**FIGURE 1:**
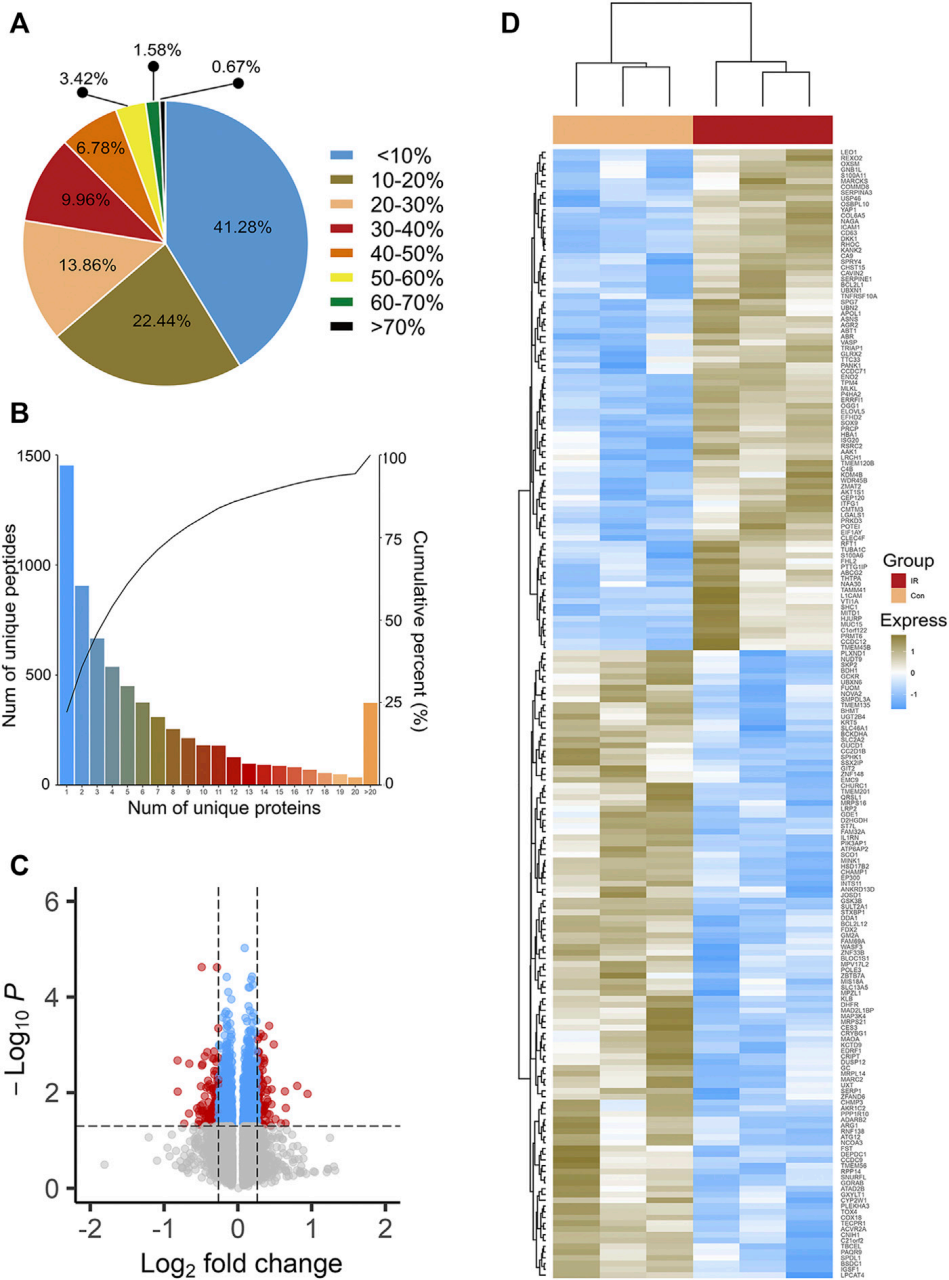
Sequence coverage distribution **(A)** and unique peptides number distribution **(B)** of proteins. **(C)** is the volcano plots of Insulin resistant HepG2 cells (IR) vs its parental HepG2 cells (CON). The most statistically significant proteins were displayed at the top of area, with upregulated proteins in red dots and downregulated proteins in green dots. Black dots represent no significant differences. **(D)** is the hierarchy clustering results expressed in tree heat maps.

A total of 118,722 peptides and 6,574 proteins were identified by iTRAQ-mass spectrometry (Supplementary Table S1). These proteins were identified with high sequence coverage, and 58.72% of the proteins had more than 10% of the sequence coverage (Figure 1A). More than 50% of proteins covered three or more peptides, which also indicated that the identified proteins had good sequence coverage (Figure 1B).

Based on two criteria (1.2-fold increase or 0.83-fold decrease and *p* < 0.05), a total of 196 proteins, including 109 downregulated proteins and 87 upregulated proteins, were identified (Supplementary Table S2) as DEPs. Statistical results of protein quantification were shown in the volcano plot (Figure 1C). Simultaneously, cluster analysis was performed on these DEPs and the result was showed in Figure 1D. All the results indicated that the biological repeats in the IR and Control groups were with good quality.

### COG and KOG Function Annotation

**FIGURE 2:**
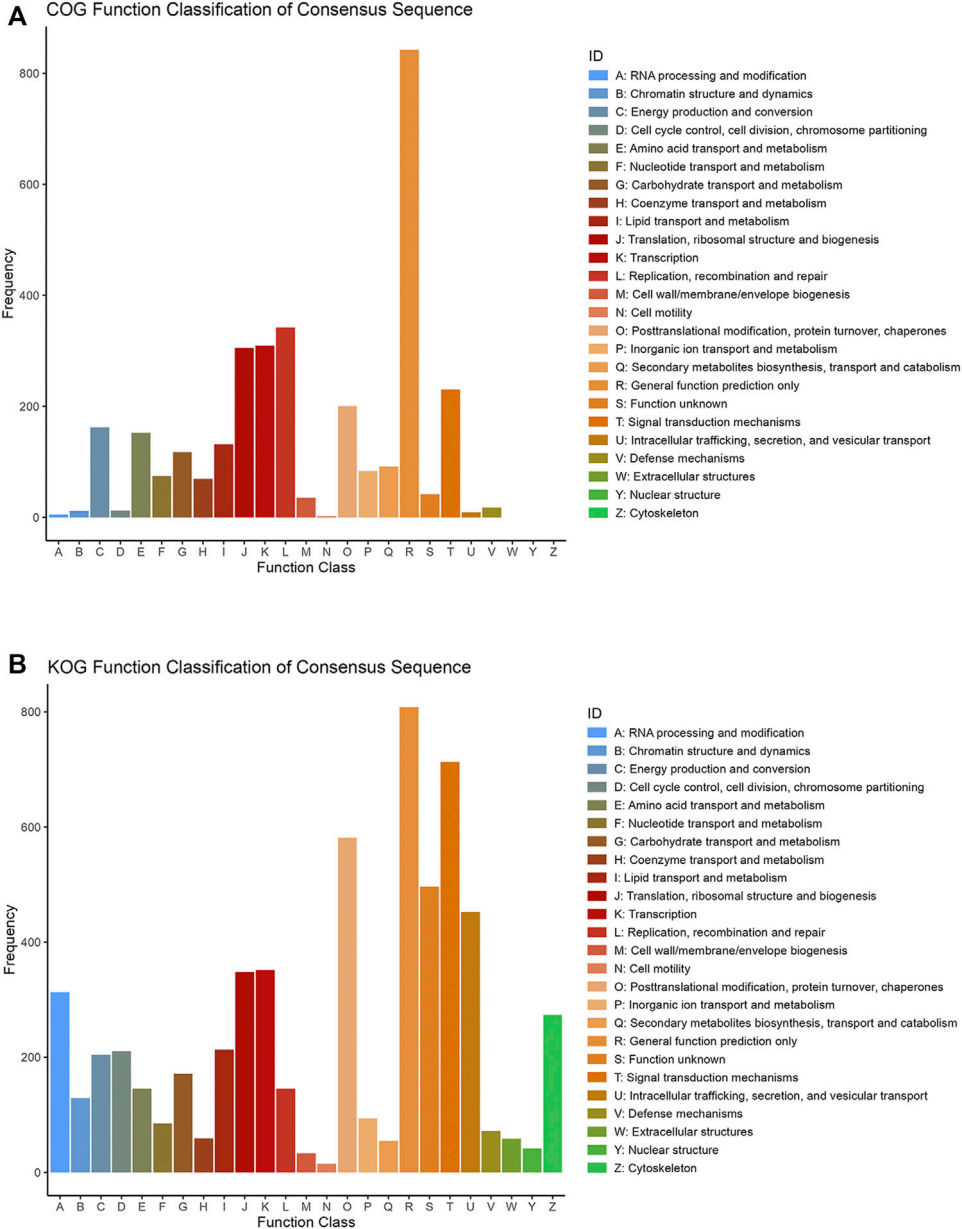
COG **(A)** and KOG **(B)** function classification of consensus sequence. The *X* axis is the classification content, and the *Y* axis is the number of proteins.

COG database was a tool for genome-scale analysis of protein functions and evolution. In present study, all the DEPs were classified into 19 COG clusters including RNA processing and modification, amino acid transport and metabolism, energy production and conversion, nucleotide transport and metabolism, carbohydrate transport and metabolism, etc. Proteins were mostly annotated and classified in “General function prediction only” (R), ‘Replication, recombination and repair’ (L), ‘Transcription’ (K), “Translation, ribosomal structure and repair(J)” etc. (Figure 2A).

KOG database was employed to classify protein orthologs (Figure 2B). Except the ‘general function prediction only’ (R), KOG noticed more proteins under the classification items of ‘Signal transduction mechanisms’ (T), ‘Posttranslational modification, protein turnover, chaperones’ (O), ‘Function unknown’ (S) and ‘Intracellular trafficking, secretion, and vesicular transport’ (U). Indicating that these differentially expressed proteins played more roles in posttranslational modification of protein transporter chaperones, signal transduction and some unknown functions in eukaryotes.

### Functional Analysis

**FIGURE 3:**
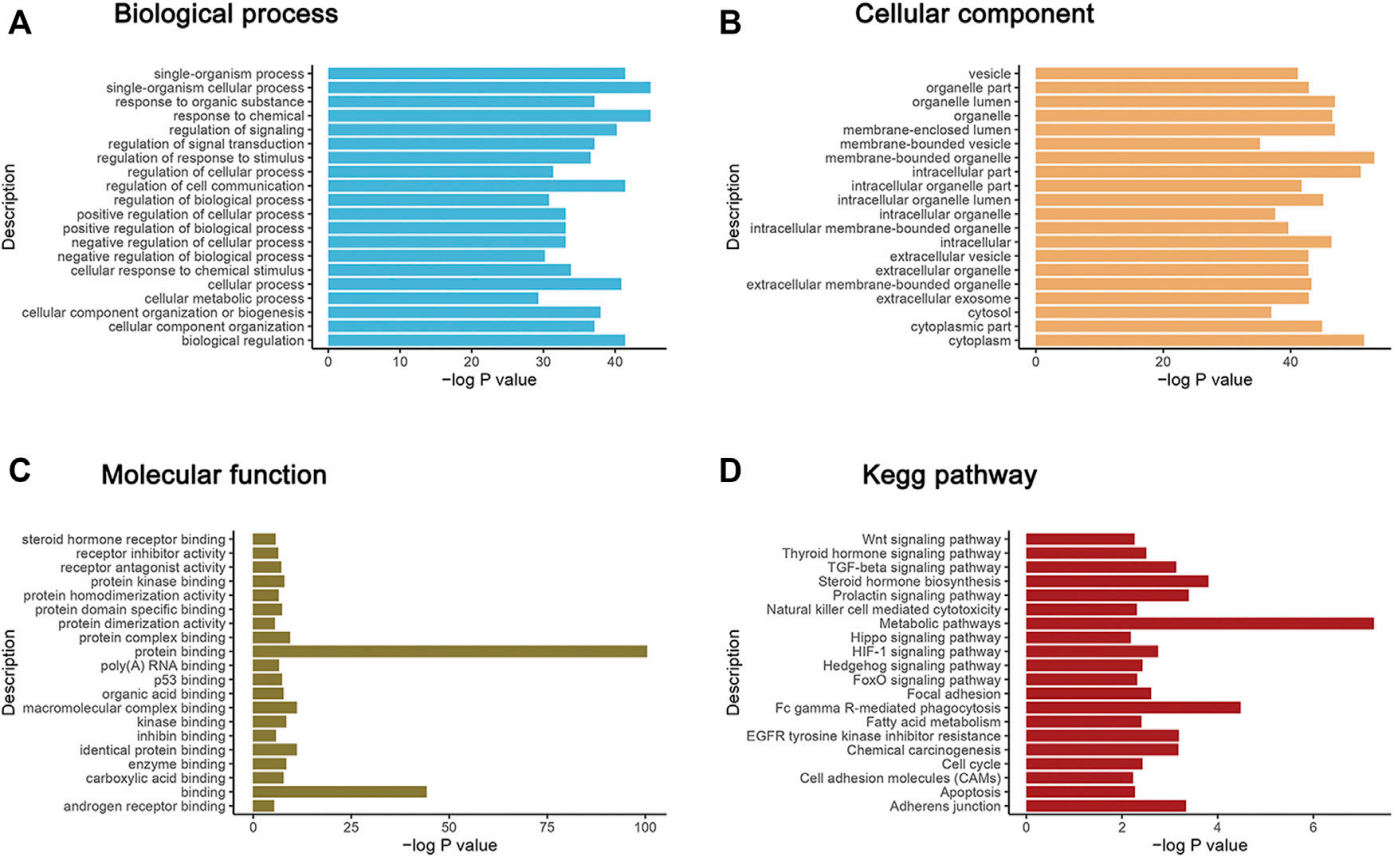
GO functional enrichment analysis and KEGG annotation enrichment analysis of DEPs. The *X* axis in the graph shows the -log10 (*p* value). The *Y* axis is enrichment to GO function classification and KEGG pathway functional classification. **(A–C)** represent the biological process (BP), cellular components (CC) and molecular function (MF) three categories. **(D)** is the KEGG annotation enrichment analysis of DEPs.

Based on GO database, the top 20 of GO terms enriched from the dysregulated proteins were response to chemical, single-organism cellular process, biological regulation, single-organism process, regulation of cell communication etc (biological process, BP); membrane-bounded organelle, cytoplasm, intracellular part, organelle lumen, membrane-enclosed lumen etc. (cellular component, CC); protein binding, binding, identical protein binding, macromolecular complex binding, protein complex binding etc (molecular function, MF) (Figures 3A–C).

Also, KEGG analysis were performed on the DEPs to enrich different pathways. Most of these enriched pathways were related with cancer metabolism, immunity, proliferation and metastasis including Wnt pathway, Hippo pathway, p53 pathway, TGF-beta pathway, HIF-1 pathway, Hedgehog pathway, Adherens junction, Apoptosis, etc. Top20 enriched KEGG pathways were shown in Figure 3D. Among which, YAP1, SOX9 and GSK3β act as key nodes with outstanding performance. They appeared frequently in most of tumor related pathways and linked them tightly.

**FIGURE 4:**
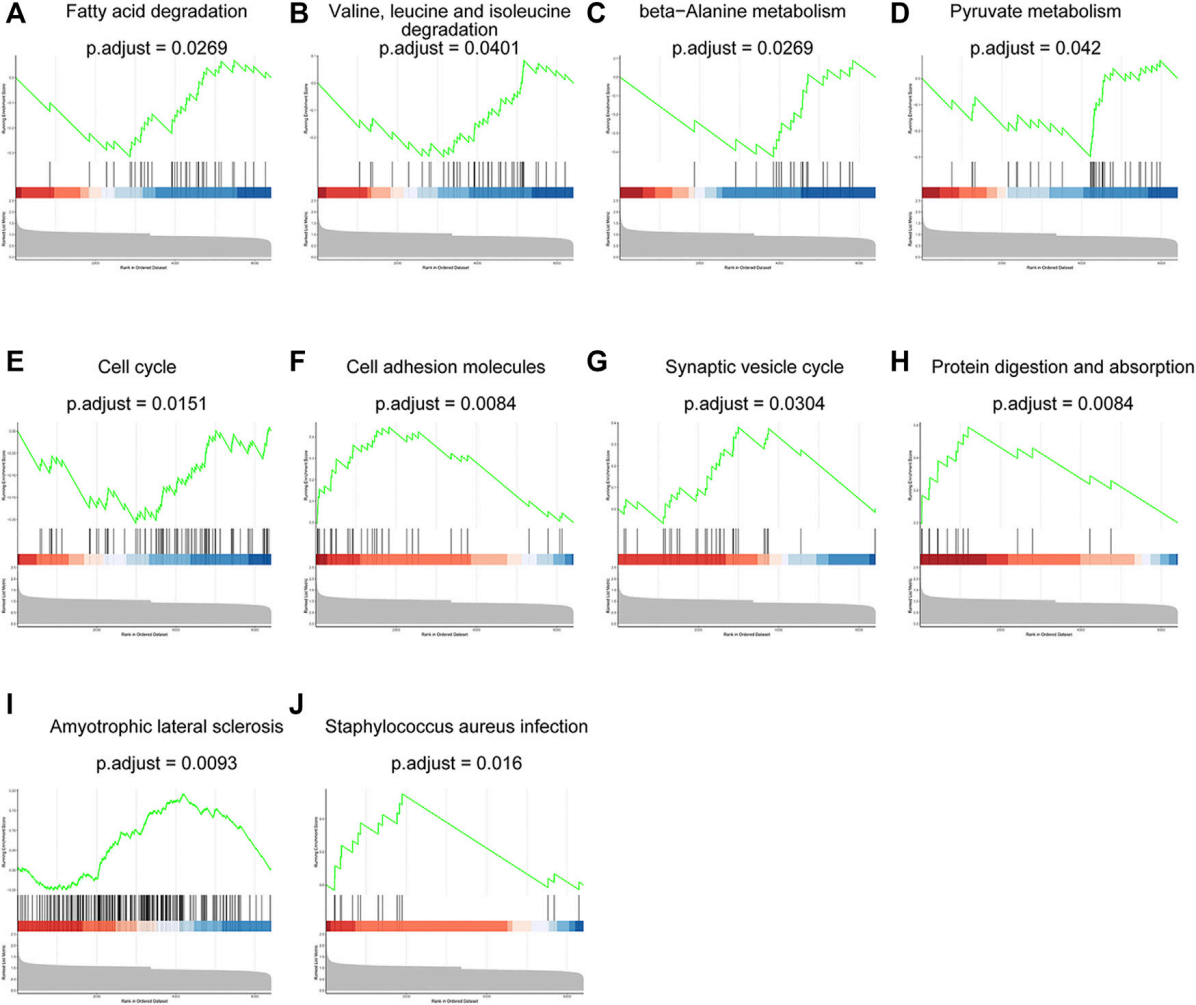
The results of GSEA analysis showed ten dysregulated proteins-related pathways. Fatty acid degradation **(A)**, valine, leucine and isoleucine degradation **(B)**, beta-Alanine metabolism **(C)**, pyruvate metabolism **(D)**, cell cycle **(E)**, cell adhesion molecules **(F)**, synaptic vesicle cycle **(G)**, protein digestion and absorption **(H)**, amyotrophic lateral sclerosis **(I)** and staphylococcus aureus infection **(J)**.

GSEA analysis was employed to illustrate the impacts from proteins with less significant fold changes and *p* value but exerted effective influence on biological function. These dysregulated proteins were enriched in 10 pathways which were shown in Figure 4. Among them, most processes were related with protein digestion and metabolism and small molecules, such as protein digestion and absorption, valine, leucine and isoleucine degradation, beta-Alanine metabolism, pyruvate metabolism, fatty acid degradation. Other processes were involved in cancer metabolic, signal communication, adhere and metastasis including cell adhesion molecules, synaptic vesicle cycle and cell cycle. The results were consistent with the features of our insulin resistant HCC model (Liu et al., 2016; Li et al., 2018; Li et al., 2020).

### Survival Analysis

**FIGURE 5:**
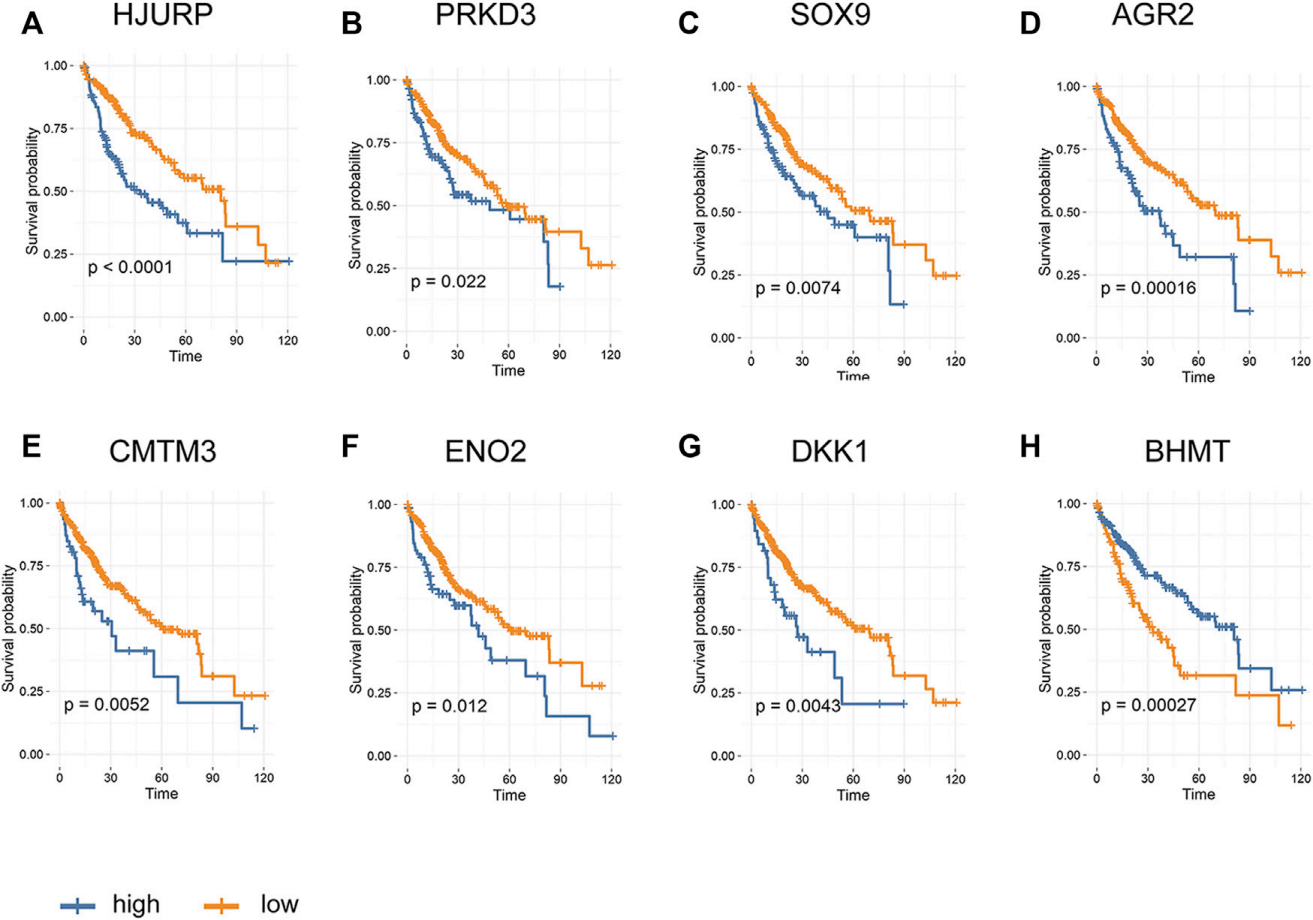
Kaplan-Meier survival analysis based on proteins expression in HCC **(A)** HJURP; **(B)** PRKD3 **(C)** SOX9; **(D)** AGR2 **(E)** CMTM3; **(F)** ENO2 **(G)** DKK1; **(H)** BHMT.

Of the 196 differentially expressed proteins, 54 were associated with prognosis as confirmed by univariate Cox regression analysis. Among which, eight proteins reported to have close relationship with tumor were chosen to further determine the greatest potential prognosis ability. A Kaplan-Meier test was used to identify these eight proteins. The results showed that higher expression of HJURP (*p* < 0.01), PRKD3 (*p* < 0.05), SOX9 (*p* < 0.01), AGR2 (*p* < 0.01), CMTM3 (*p* < 0.01)), ENO2(*p* < 0.05) and DKK1(*p* < 0.01) could bring lower rate of survival, whereas up-regulation of BHMT (*p* < 0.01) represented better life expectancy (Figure 5), indicating that the dysregulated proteins existing in IR hepatoma cells not only participated in malignant phenotype of HCC, but also closely related to survival in patient with liver cancer.

### PPI Network Analysis

**FIGURE 6:**
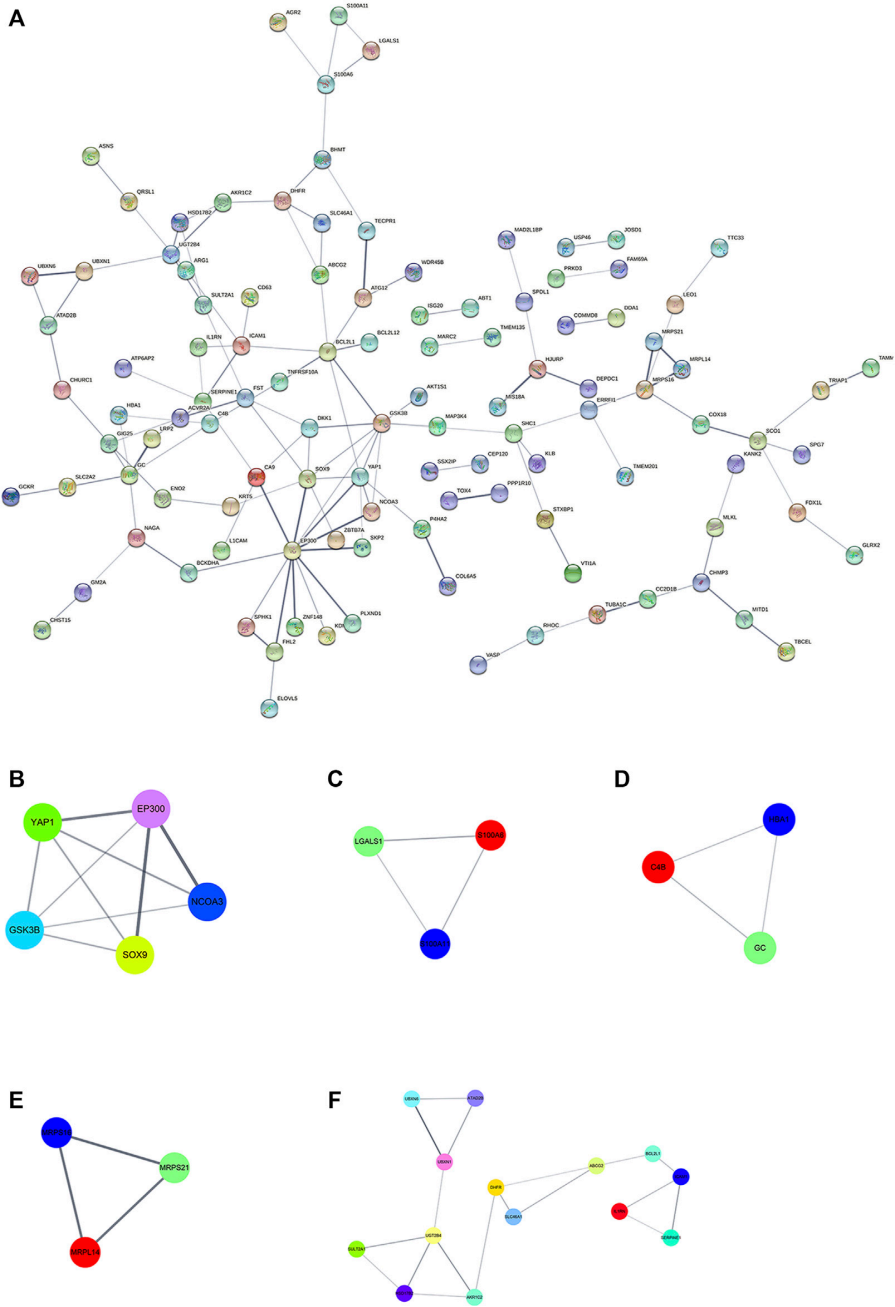
PPI network and module analysis **(A)** PPI network for DEPs; **(B)** critical module one in PPI network **(C)** critical module two in PPI network; **(D)** critical module three in PPI network **(E)** critical module four in PPI network; **(F)** critical module five in PPI network. Different colored lines represent different evidences. The straight line (edge) represents the interaction between proteins. The thicker the line, the stronger the interaction between them.

To further illustrate the potential molecular function of DEPs in HepG2/IR cells, a protein-protein co-expression network were constructed for the DEPs by using STRING database and Cytoscape software. Hiding disconnected nodes, this PPI network contained a total of 194 nodes and 128 edges (Figure 6A). Then the hub proteins were screened by computing degree and betweenness, and the top 10 proteins were obtained as following: EP300, GSK3β, BCL2L1, SOX9, YAP1, GC, ICAM1, MRPS16, SERPINE1. Subsequently, the co-expression network was further analyzed to detect potential critical modules by using plug-in MCODE in Cytoscape, and five significant modules were determined. Module 1 with the highest score included five nodes and nine edges (Figure 6B), module 5 with most nodes and edges consisted of 14 nodes and 18 edges (Figure 6F), and other three modules had three nodes and edges (Figures 6C,D,F). The GO and pathway analyses showed that the proteins from module one were mainly enriched in function of cancer signal transduction, whereas the proteins in module five were significantly enriched in function of autophagy regulation (Supplementary Table S3).

### Validation of the Selected Proteins by PRM and Western Blot

**FIGURE 7:**
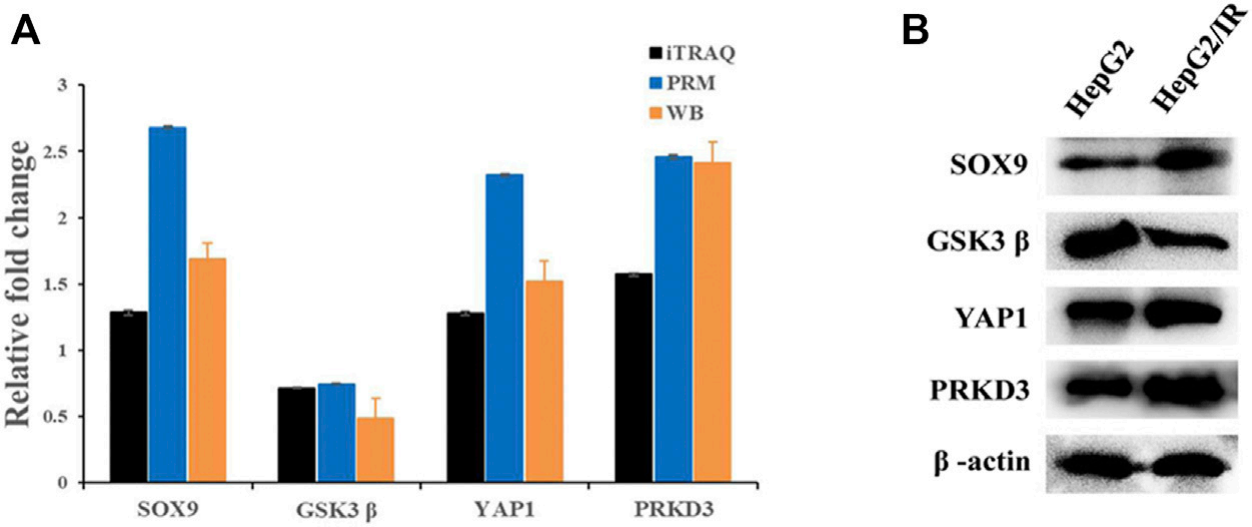
The comparison of protein expression detected by iTRAQ and PRM **(A)** and Western blot **(B)**. Relative fold change means the ratio of Insulin resistance group and Control group.

PRM is currently a major approach to achieve the absolute quantification of the target proteins by selectively detecting specific peptides. To verify the accuracy of the proteomics data, four differential expressed proteins with key functions were chosen to determine the expression levels by PRM and Western blot quantitative analysis, including Yes1 associated transcriptional regulator (YAP1), SRY-box transcription factor 9(SOX9), protein kinase D3 (PRKD3) and glycogen synthase kinase three beta (GSK3β). The expression levels of these proteins were significantly changed during the iTRAQ Profiling and were closely related to malignant features such as cell proliferation, metabolism, immunity and tumor. Consisted with the results of iTRAQ, all the validation results showed a same trend in both Western blot and PRM analysis The validation results of RRM showed that the relative fold changes (Insulin resistant group/Control group) with PRKD3 (2.46), SOX9 (2.68) and YAP1 (2.32) were upregulated evidently compared with iTRAQ analysis, while GSK3β (0.74) was closed to iTRAQ analysis (Figure 7A). The qualification of Western blot showed that the relative fold changes were PRKD3 (2.41), SOX9 (1.69), YAP1 (1.52) and GSK3β (0.48) separately (Figures 7A,B), indicating that the iTRAQ results were credible for further analysis.

## Discussion

Compared to traditional transcriptomics or genomics which assess only the messenger and interpreted information at the gene level, proteomics describes molecular responses more directly through collecting information from all proteins encoded by the genome with high comprehensiveness and accuracy. Based on that, it can facilitate better understanding human physiological and pathological processes (Xia et al., 2020). iTRAQ, as a novel quantitative proteomic approach, is widely used in biological research and has been proven to be a useful technique for identifying and detecting DEPs among different samples (Yang et al., 2020).

In the present study, iTRAQ technology was employed and LC-MS/MS and Western blot were combined to dissect the DEPs between insulin resistant and their parental HCC for the first time. Among the 6,645 proteins identified, 109 were obviously downregulated and 87 were clearly upregulated, on a basis of a fold change <0.83 or >1.2 with a *p* value <0.05 for the differentially expressed proteins. All of the 196 DEPs were further analyzed using GO functional annotation and enrichment analysis, KEGG pathway enrichment analysis, and protein-protein interaction network analysis.

Our previous studies suggested that IR hepatoma cells showed malignant biological phenotypes, including enhanced proliferation, invasion and drug resistance. These functional changes may be related to some dysregulated proteins (Liu et al., 2016; Li et al., 2018; Li et al., 2020). Biological functions and pathway analysis such as GO, KEGG and GESA further indicated that these disturbed pathways enriched by DEPs were highly involved in tumorigenesis and development. These pathways included adherens junction, TGF-beta pathway, Wnt pathway, Hippo pathway, mTOR pathway etc. (Ediriweera et al., 2019; Li et al., 2021; Parsons et al., 2021; Sabbadini et al., 2021). PPI network showed that three hub proteins, SOX9, YAP1 and GSK3β, were located in the key positions, and all of them were involved in Wnt and Hippo signaling pathways (Stewart, 2014; Ediriweera et al., 2019; Li et al., 2021). Besides that, survival analysis for HCC patients from the TCGA database revealed that high expression of SOX9 and PRKD3 were strongly associated with reduced patient survival rate.

Based on the above analysis, four differentially expressed candidate proteins which significantly different expressed and closely related to malignant features were selected for validation. Since the PRM technology is based on high-resolution and high-precision mass spectrometers and the results of PRM analysis are undoubtedly technically reliable (Li et al., 2019a), we used the PRM analysis and Western blot assay to validate these four candidate proteins. Both methods verified that the expression levels of PRKD3, YAP1 and SOX9 were upregulated, while GSK3β showed the opposite trend in HepG2/IR cells. The high consistency among the results of iTRAQ, PRM and Western blot indicated the accuracy and reproducibility of our study, which also provided some reliable and potential targets for overcoming insulin resistance in HCC.

PRKD3, a member of the calcium/calmodulin kinase superfamily, act as the downstream of G protein coupled receptor and tyrosine kinase receptor. PRKD3 was reported to play a certain cancer-promoting role in specific types of tumors. Liu et al. declared that PRKD3 acted as a stimulating factor promoting proliferation of breast cancer cells (Liu et al., 2020). Huck et al. pointed out that although the expression of PRKD3 was not obvious in some breast cancer, it was significantly increased in highly aggressive triple negative breast cancer and negatively regulated by estrogen (Huck et al., 2014; Borges et al., 2015). Other studies had confirmed that PRKD3 was highly expressed in prostate cancer and played a role in regulating tumor cell migration and invasion (Chen et al., 2008; Zou et al., 2012). The cancer-promoting effect of PRKD3 in HCC had also been studied. Yang et al. suggested that high expression of PRKD3 in HCC was closely related to poor prognosis (Li et al., 2019b). The molecular mechanism of PRKD3 in promoting tumorigenesis is not yet fully understood. Huck pointed out that PRKD3 promoted growth of triple negative breast cancer cells by activating mTORC1-S6K1 pathway. Chen suggested that PRKD3 could promote prostate cell proliferation by activating downstream AKT and ERK1/2 molecules, and the absence of PRKD3 could lead to the arrest of cell cycle G_0_/G_1_ phase of prostate cancer cell line (Chen et al., 2008). Li et al. confirmed that the protein could promote cancer by upregulating lipid synthesis (Li et al., 2019b). Hippo/YAP1 pathway has emerged as a universal governor and therapeutic target in cancers. The functions of Hippo/YAP1 pathway involve organ size regulation, tissue regeneration, tumorigenesis and G protein coupled signal pathway. YAP1, as an oncoprotein, is inhibited by Hippo pathway in most tumor cells (Moroishi et al., 2015). Some studies suggested that PRKDs could phosphorylate serine 127 and 397 sites of YAP1 protein, promoted the aggregation of YAP1 protein in cytoplasm, upregulated the mRNA expression of YAP-TEAD regulatory genes *CTGF* and *AREG* and promoted the proliferation of upper intestinal small skin cells, indicating that PRKDs were related to the regulation of YAP pathway activity and the promotion of cell growth (Wang et al., 2016). However, the relationship between Hippo/YAP1 pathway and PRKD3 and its fuction in tumorigenesis and development have not been reported so far. The iTRAQ results screened out that the protein level of PRKD3 and YAP1 were both increased in insulin resistance hepatocellular carcinoma cells. Our unpublished data proved that the expression of YAP1 was changed in the same direction as PRKD3. However, the regulation mechanisms between PRKD3 and YAP1 still need to be further studied.

SOX9, a member of the SOX family of transcription factors with high mobility group box DNA binding and transactivation domains, participates in a wide range of functions, including development and progression of various diseases. It was confirmed that SOX9 could interact with various partners and showed stimulating or suppressing activities in different cell types (Jana et al., 2020). The upregulated of SOX9 was associated with the tumor occurrence, invasion, metastasis, and poor patient survival rate. In lung cancer, highly expressed SOX9 promoted cell proliferation and inhibited apoptosis by activating Wnt/β-catenin signaling through phosphorylation of GSK3β (Guo et al., 2018; Huang et al., 2019). The iTRAQ result of this study suggested that SOX9 was increased in IR HCC cells in contrast to their parental cells, which provides another possible role of SOX9 in insulin resistance regulation, although the underlying mechanisms remain to be further studied.

GSK3β which belongs to mitogen-activated protein (MAP) kinase superfamily is a multifunctional serine/threonine protein kinase. Aberrant expression and activity of GSK3β contributes to the pathogenesis and progression of common diseases such as glucose intolerance and cancer. Deregulated GSK3β also takes part in tumor cell survival, resistance of apoptosis, proliferation, migration and invasion, as well as sustaining cancer stemness (Domoto et al., 2020). A crucial pathway that is regulated by GSK3β is Wnt/β-catenin pathway which plays an important role in proliferation as well as EMT (epithelial-mesenchymal transition) (Duda et al., 2020). Compared to insulin sensitive HepG2 cells, the expression level of SOX9 in HepG2/IR cells was significantly increased in this study, while lower level of GSK3β was also observed in insulin resistant HepG2 cells. Nevertheless, whether SOX9 and GSK3β have reciprocal regulatory relationship, how these regulations occur, and how they participate in insulin resistance are still not clear.

In summary, our present study identified DEPs and indicated some potential signaling pathways that related to the insulin resistant HCC, which might provide possible targets for alleviating drug resistance. Further studies need to be done to elucidate the functions, related signaling pathways and correlations of these DEPs.

## Data Availability

The datasets presented in this study can be found in online repositories. The names of the repository/repositories and accession number can be found below: http://proteomecentral.proteomexchange.org, Accession Number PXD031820.
